# Associations between Family-Related Factors, Breakfast Consumption and BMI among 10- to 12-Year-Old European Children: The Cross-Sectional ENERGY-Study

**DOI:** 10.1371/journal.pone.0079550

**Published:** 2013-11-25

**Authors:** Wendy Van Lippevelde, Saskia J. Te Velde, Maïté Verloigne, Maartje M. Van Stralen, Ilse De Bourdeaudhuij, Yannis Manios, Elling Bere, Froydis N. Vik, Nataša Jan, Juan M. Fernández Alvira, Mai J. M. Chinapaw, Bettina Bringolf-Isler, Eva Kovacs, Johannes Brug, Lea Maes

**Affiliations:** 1 Department of Public Health, Ghent University, Ghent, Belgium; 2 Department of Epidemiology and Biostatistics, VU University Medical Center, EMGO Institute for Health and Care Research, Amsterdam, The Netherlands; 3 Department of Movement and Sport Sciences, Ghent University, Ghent, Belgium; 4 Department of Public and Occupational Health, VU University Medical Center, EMGO Institute for Health and Care Research, Amsterdam, The Netherlands; 5 Department of Nutrition and Dietetics, Harokopio University, Athens, Greece; 6 Faculty of Health and Sport, University of Agder, Kristiansand, Norway; 7 Slovenian Heart Foundation, Ljubljana, Slovenia; 8 Instituto Aragonés de Ciencias de la Salud, University of Zaragoza, Zaragoza, Spain; 9 Swiss Tropical and Public Health Institute, Department of Epidemiology and Public Health; 10 University of Basel, Basel, Switzerland; 11 Department of Paediatrics, University of Pécs, Pécs, Hungary; University of Missouri-Kansas City, United States of America

## Abstract

**Objective:**

To investigate associations of family-related factors with children’s breakfast consumption and BMI-z-score and to examine whether children’s breakfast consumption mediates associations between family-related factors and children’s BMI-z-score.

**Subjects:**

Ten- to twelve-year-old children (n = 6374; mean age = 11.6±0.7 years, 53.2% girls, mean BMI-z-score = 0.4±1.2) and one of their parents (n = 6374; mean age = 41.4±5.3 years, 82.7% female, mean BMI = 24.5±4.2 kg/m^2^) were recruited from schools in eight European countries (Belgium, Greece, Hungary, the Netherlands, Norway, Slovenia, Spain, and Switzerland). The children self-reported their breakfast frequency per week. The body weight and height of the children were objectively measured. The parents responded to items on family factors related to breakfast (automaticity, availability, encouragement, paying attention, permissiveness, negotiating, communicating health beliefs, parental self-efficacy to address children’s nagging, praising, and family breakfast frequency). Mediation analyses were performed using multi-level regression analyses (child-school-country).

**Results:**

Three of the eleven family-related variables were significantly associated with children’s BMI-z-score. The family breakfast frequency was negatively associated with the BMI-z-score; permissiveness concerning skipping breakfast and negotiating about breakfast were positively associated with the BMI-z-score. Children’s breakfast consumption was found to be a mediator of the two associations. All family-related variables except for negotiating, praising and communicating health beliefs, were significantly associated with children’s breakfast consumption.

**Conclusions:**

Future breakfast promotion and obesity prevention interventions should focus on family-related factors including the physical home environment and parenting practices. Nevertheless, more longitudinal research and intervention studies to support these findings between family-related factors and both children’s breakfast consumption and BMI-z-score are needed.

## Introduction

Overweight and obesity in youths have increased during the past decades and are associated with different physical and psychosocial health problems. [1] Overweight is caused by a long-term positive energy balance occurring when the energy intake outweighs the energy expenditure. [2] Breakfast consumption in children and adolescents was found to be inversely related to the Body Mass Index (BMI) and overweight in both cross-sectional [3]–[7] and longitudinal studies. [8], [9] Eating breakfast has been suggested to potentially prevent snacking and the consumption of energy-rich foods. [3], [6], [7], [10] Moreover, regular breakfast consumption has been associated with overall dietary quality and nutritional profiles in school-aged children [3], [7], [8] and with improved cognitive performance. [6] However, whether breakfast as such is of great importance or whether skipping breakfast is an indicator of an overall irregular meal and eating pattern is unclear. Despite the potential importance of breakfast consumption, the prevalence rates of breakfast skipping among children and adolescents has increased in the past few decades. [11]–[13] In addition, the prevalence of regular breakfast consumption tends to decrease as children grow older. [3] Therefore, interventions promoting breakfast consumption during childhood are urgently needed. To develop effective interventions, knowledge about the underlying factors is important.

It is well known that parents play a major role in the development of healthy eating habits in their children through a variety of mechanisms including role modelling a healthy diet, the availability and accessibility of nutritious foods at home, and the development of attitudes, values, and preferences. [14]–[16] Systematic reviews on family correlates of children’s breakfast consumption found that the parents’ breakfast intake was positively associated with the breakfast intake of their children. [17], [18] To date, only a few studies have examined some associations between physical (e.g., availability, and accessibility), sociocultural (e.g., support) and political (e.g., rules) family factors and breakfast consumption. [17], [18] However, to our knowledge, no studies are available that have examined a wide range of family-related factors at the same time or their relation with children’s breakfast intake.

In the recent “EuropeaN Energy balance Research to prevent excessive weight Gain among Youth” (ENERGY)-project, [19] a wide range of physical and sociocultural family factors related to children’s breakfast consumption were measured. [20] This project provided the opportunity to explore the associations between family-related factors and children’s breakfast consumption. In addition, given the evidence that skipping breakfast is a predictor of overweight, we also wanted to investigate how family-related factors concerning breakfast consumption and children’s breakfast consumption relate to children’s BMI-z-score, and whether children’s breakfast consumption influences the relation between the family-related factors and children’s BMI-z-score. To our best knowledge, no studies have examined the relation between family-related variables and children’s BMI-z-score or investigated the mediating effect of children’s breakfast intake on these relations.

The purposes of this study were the following: (i) study relations between the family-related variables related to breakfast and children’s breakfast consumption, (ii) investigate associations between the family-related variables related to breakfast and children’s BMI-z-score, and (iii) determine whether children’s breakfast consumption acts as a mediator of the association between the family-related variables related to breakfast and children’s BMI-z-score.

## Methods

The ENERGY-project included a school-based cross-sectional survey assessing overweight, obesity and energy balance-related behaviours (EBRBs: modifiable energy intake and energy expenditure behaviours such as dietary, physical activity and sedentary behaviours) and their determinants across eight European countries (Belgium, Greece, Hungary, the Netherlands, Norway, Slovenia, Spain, and Switzerland). The survey entailed anthropometric measurements, a child questionnaire, a parent questionnaire, a school-staff questionnaire and school observations to measure overweight indices, EBRBs and potential individual and environmental correlates of these behaviours. A description of the design and conceptual framework of the ENERGY-project19 and an extensive description of the design, procedures, and methodology of the ENERGY school-based survey [20] are provided elsewhere. The data collection manual and survey questionnaires for the ENERGY cross-sectional survey are available online at http://projectenergy.eu. Ethical approval was obtained from Medical Ethical review committees in all participating countries. In Belgium, the survey was approved by the Medical Ethics Committee of the University Hospital Ghent; in Greece the survey was approved by the Bioethics Committee of Harokopio University; in Hungary the survey was approved by the Scientific and Ethics Committee of Health Sciences Council; in the Netherlands the survey was approved by the Medical Ethics Committee of the VU University medical center; in Norway the survey was approved by the National Committees for Research Ethics in Norway; in Slovenia the survey was approved by the National Medical Ethics Committee of the Republic of Slovenia; in Spain the survey was approved by Clinical Research Ethics Committee of the Government of Aragón; in Switzerland the survey was approved by the ethic committees of Aargau, Basel, Bern and St. Gallen. Furthermore, research permission was, if necessary, obtained from local school authorities (local school boards and/or headmasters).

### Sampling and Participants

The survey was conducted between March and December 2010 in eight European countries among 10- to 12-year old children. Based on previous cross-European studies (e.g. Pro Children [21]), a minimum sample of 1000 schoolchildren per country and one parent/caretaker for each child was aimed for. National sampling was used in Greece, Hungary, the Netherlands and Slovenia, whereas schools from specific regions were sampled in Belgium (i.e. Flanders), Norway (i.e., southern regions), Spain (i.e., Aragón) and Switzerland (i.e., the German speaking region). Due to the differences in population distribution within the different regions and countries, the sampling of schools was random, multi-staged, and stratified by the degree of urbanisation. More extensive information about the recruitment procedure can be found in van Stralen and colleagues [20].

A school recruitment letter was sent to the headmasters or principals of the sampled schools, followed by a personal call. Following the school’s agreement, the parents received a letter explaining the study purpose and were asked for written consent for their children’s and their own participation.

### Measures

The measurements were conducted according to standardised protocols. [20] The children completed questionnaires during school time. In addition, anthropometrical measurements were conducted. The children received the parent questionnaire in a closed envelope to take home for completion by one of their parents. Detailed information regarding the procedures, training of research staff, development of questionnaires, [20] and test-retest reliability and construct validity [22], [23] of the child and parent questionnaires are published elsewhere.

#### Child anthropometric measurements

Body height and weight were measured by trained research assistants. The children were measured in light clothing without shoes. Body height was measured with a Seca Leicester Portable stadiometer with an accuracy of 0.1 cm, and weight was measured with a calibrated electronic scale SECA 861 with an accuracy of 0.1 kg. Two readings of each measurement were obtained. If the two readings differed by more than 1%, a third measurement was taken. All three measurements were recorded and the outlier was excluded during the data cleaning process. Subsequently the mean of the two nearest measures was calculated. BMI-for-age z-scores (BMI-z-scores) were calculated based on the WHO criteria [24].

#### Child breakfast consumption

Breakfast was defined as items consumed within two hours after getting up in the morning during school days. In weekends, breakfast was defined as having something to eat and/or drink before 11 a.m. Breakfast consumption was assessed by two questions asking the children on how many schooldays per week [0–5] and how many weekend days [0–2] they normally had breakfast. Breakfast frequency per week [0–7] was calculated by adding up the answers to the two questions. These items were validated in a separate study and were found to be sufficiently reliable and valid compared with a cognitive interview [22].

#### Parental measures (demographics, family-related factors)

In the parent questionnaire, demographics and self-reported levels of parental breakfast behaviour and other family-related variables related to breakfast were assessed.

Age, weight, height, and educational level were assessed using one question. Parental education was categorised as being high (at least one parent with more than 14 years of education) or low (both parents having less than 14 years of education), which in this international dataset approximately distinguishes families with at least one caregiver who has completed medium or higher vocational, college or university training from other families. [25] The BMI (weight/height squared) was calculated from the self-reported height and weight of the parent who completed the questionnaire.

Questions on parental breakfast consumption were similar to those in the child questionnaire; breakfast consumption was assessed by frequency questions referring to a general week. These items were also validated in a separate study and were found to be sufficiently reliable and valid compared with a cognitive interview. [23] Table 1 shows the exact formulations of the questionnaire items to measure the family-related factors (i.e., automaticity, availability, encouragement, paying attention, allowing to skip breakfast, negotiating, communicating health beliefs, parental self-efficacy, praising, eating breakfast together) and their psychometric characteristics. The family-related questionnaire items were based on and informed by the Pro Children and ENDORSE parent questionnaires. [26], [27] The items had a five-point answering format. Exploratory factor analyses showed that two items (i.e. (1) if I prohibit my child from skipping breakfast, (s)he tries to skip it anyway; (2) if I prohibit my child from skipping breakfast, I find it difficult to stick to my rule(s) if (s)he starts negotiating) could be collapsed into the subscale ‘parental self-efficacy to manage the child’s breakfast behaviour’ (Cronbach’s alpha>0.70). The subscale and all other singular family-related items were used as independent variables in the model.

**Table 1:** Formulations of the questionnaire items and the psychometric characteristics.

Factor	Question item	Response alternatives
Automaticity	Eating breakfast is something I do without even reallythinking about.	−2 = fully disagree – 2 = fully agree
Availability	There are breakfast products (milk, cereals, bread etc)available at home for my child.	0 = never – 4 = always
Encouragement	I encourage my child to have breakfast.	0 = never – 4 = always
Paying attention	I pay attention what kind of products my child is eatingfor breakfast.	0 = never – 4 = always
Allowing to skip breakfast	My child is allowed to skip breakfast.	0 = never – 4 = always
Negotiating	I negotiate with my child on how much breakfastproducts (s)he has to eat and/or drink.	0 = never – 4 = always
Communicating health beliefs	How often do you tell your child that eatingbreakfast is good for you.	0 = never – 4 = always
Parental self-efficacy (2 items, Cronbach’salpha >0.70)	1. If I prohibit my child from skipping breakfast,(s)he tries to skip it anyway.	0 = never – 4 = always
	2. If I prohibit my child from skipping breakfast, I findit difficult to stick to my rule(s) if (s)he starts negotiating.	
Praising	I praise my child if (s)he eats breakfast.	0 = never – 4 = always
Eating breakfast together	How often do you eat breakfast with yourparents/care givers?	0 = never – 7 = every day

### Statistical Analyses

Preliminary analyses consisting of the descriptive statistics of sample characteristics and key variables were conducted using SPSS (version 15). The normality of the key variables was checked. Although the outcome variable showed a skewed distribution, the distribution of the residuals was acceptable. Therefore, the untransformed outcome variable was used. We used a complete cases design and therefore included only children who had valid measurements for breakfast intake, height, and weight, but not necessarily for all potential correlates of breakfast consumption.

Multilevel linear regression analyses were performed to assess associations between the family-related variables and both children’s breakfast consumption, and the BMI-z-score of the children using MLwiN version 2.22 (three-level random intercept model: children nested within schools nested within countries). In addition, we tested whether associations between the family-related variables and children’s BMI-z-score were mediated by children’s breakfast consumption by applying the product-of-coefficient method [28].

**Figure 1:**
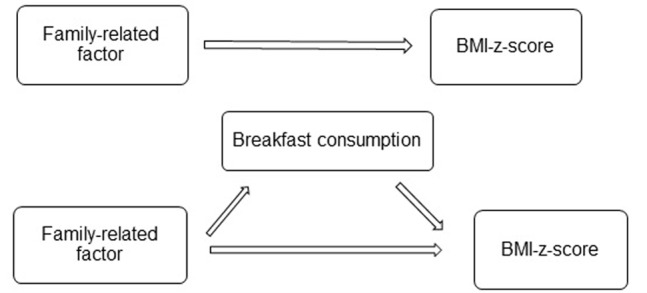
Mediation model with family-related factor (independent variable), breakfast consumption (mediator), BMI-z-score (dependent variable) and the different coefficients. c- coefficient: estimate of the association between family-related factor item and BMI-z-score. c’-coefficient: estimate of the association between family-related factor and BMI-z-score, adjusted for children’s breakfast consumption (mediator). a –coefficient: estimate of the association between family-related factor and children’s breakfast consumption (mediator). b_coefficient: estimate of the association between children’s breakfast consumption (mediator) and children’s BMI-z-score, adjusted for family-related factor.

First, associations between each family-related variable and children’s BMI-z-score were examined (c-path). Second, associations between each family-related variable and children’s breakfast consumption (potential mediator) were studied (Action Theory test, a-path). Third, associations between children’s breakfast consumption (potential mediator) and children’s BMI-z-score (Conceptual Theory Test, b-path) adjusted for the family-related variables, and associations between each family-related variable and children’s BMI-z-score adjusted for children’s breakfast consumption were estimated (c’-path (see also figure 1).

The mediated effect was calculated by multiplying the a-coefficient with the b-coefficient using the product-of-coefficient (a coefficient*b coefficient).28 The statistical significance of the mediated effect was estimated by dividing the product-of-coefficient (a*b) by its standard error. For the calculation of the standard error, the Sobel test was used (SEab = √(a2*SEb2+b2*SEa2). Significance was set at the p<0.05 level. In addition, the proportion mediated was calculated by dividing the total mediated effect by the sum of the direct effect (c’-path) and the total mediated effect (a*b/(c’+a*b)). Mediation was considered partial when the association between the family-related variables and the BMI-z-score remained significant after adjustment for the potential mediator (c’-path). [29] In addition, according to MacKinnon, 28 a significant total effect (c-path) is not a necessary condition for mediation to occur. It can be relevant to study mediating effects of non-significant associations because it can identify the presence of unmeasured variables that suppress the association. All analyses were adjusted for children’s gender, and the parents’ age, education and BMI as these constructs were significantly associated with the outcome and potential mediators. Additionally, the need for country-specific mediation analyses was determined by examining the moderating role of country on the relation between the different family-related variables and both children’s BMI and breakfast intake: in models with children’s BMI and breakfast intake used separately as the outcome, a test for the interaction between country of residence (country of residence defined by seven dummy variables) and each studied family-related variables was conducted. Where the interaction terms were significant, separate mediation analyses per country were conducted.

## Results

### Study Characteristics

In total, 7915 children and 6512 parents across eight countries completed the cross-sectional ENERGY questionnaire from which 7625 children had valid data for breakfast consumption, height, and weight corresponding with 6374 parent questionnaires thus all analyses were conducted on the 6374 child-parent dyads. Descriptives of the demographics, behaviours and family-related variables are shown in Table 2.

**Table 2:** Descriptive statistics of the study sample (n = 6374 child-parent dyads).

	Total	Belgium	Greece	Hungary	The Netherlands	Norway	Slovenia	Spain	Switzerland
	n = 6374	n = 984	n = 1074	n = 1018	n = 855	n = 968	n = 1132	n = 1005	n = 589
	mean (SD) or %	mean (SD) or %	mean (SD) or %	mean (SD) or%	mean (SD) or %	mean (SD) or %	mean (SD) or %	mean (SD) or %	mean (SD) or %
**Demographics**									
Age child	11.6(0.7)	11.5(0.7)	11.3(0.6)	12.2(0.6)	11.6(0.7)	12.0(0.7)	11.4(0.6)	11.4(0.6)	11.6(0.8)
Age parent	41.4(5.3)	40.8(4.6)	41.5(6.0)	39.5(5.3)	42.4(4.8)	42.0(5.0)	40.6(5.0)	42.6(4.5)	42.5(6.3)
Sex (%)									
Female	53.2	54.7	55.3	55.6	50.9	53.2	53	52	47.7
Sex parent (%)									
Female	82.7	87.4	82.1	85.4	90.9	79.3	79.1	80.9	81.7
Family educational level									
at least one parent ≥14 years of education	64.9	84.2	51.6	58.1	77.8	74.8	56.2	80.5	40.5
**Anthropometrics**									
z-score Body Mass Index child	0.4(1.2)	0.1(1.1)	1.0(1.2)	0.3(1.2)	0.1(1.0)	0.2(1.0)	0.5(1.2)	0.5(1.1)	0.02(1.1)
Body Mass Index parent	24.5(4.2)	24.1(4.4)	25.2(4.6)	24.7(4.7)	24.6(4.8)	24.6(3.7)	24.7(4.2)	23.9(3.4)	23.6(3.9)
**Behaviours**									
Children’s weekly breakfast intake [0–7]	6.0(1.8)	6.2(1.6)	5.4(2.1)	5.7(1.9)	6.6(1.2)	6.4(1.4)	5.2(2.2)	6.6(1.1)	6.1(1.7)
Parents’ weekly breakfast intake [0–7]	5.7(2.2)	6.3(1.8)	4.7(2.6)	5.3(2.4)	6.6(1.4)	6.5(1.4)	2.0(2.4)	6.5(1.4)	5.7(2.2)
**Family-related factors**									
Automaticity [−2–2]	0.6(1.6)	0.7(1.5)	0.5(1.5)	1.2(1.3)	0.7(1.5)	1.2(1.3)	0.5(1.7)	0.1(1.8)	−0.1(1.6)
Availability [0–4]	3.9(0.3)	3.9(0.3)	3.9(0.4)	3.9(0.4)	4.0(0.3)	4.0(0.2)	3.9(0.3)	4.0(0.1)	3.9(0.3)
Encouragement [0–4]	3.7(0.8)	3.8(0.7)	3.7(0.8)	3.8(0.7)	3.7(1.0)	3.8(0.7)	3.6(0.8)	3.7(0.9)	3.5(1.1)
Paying attention [0–4]	3.4(0.9)	3.2(1.0)	3.7(0.7)	3.3(0.9)	3.3(0.9)	3.6(0.7)	3.1(1.0)	3.8(0.6)	3.4(0.9)
Allowing to skip breakfast [0–4]	0.8(0.9)	0.6(0.9)	1.1(1.0)	1.0(1.0)	0.3(0.5)	0.5(0.7)	1.1(1.0)	0.3(0.6)	0.9(1.1)
Negotiating [0–4]	1.4(1.3)	1.6(1.3)	2.0(1.4)	1.0(1.2)	1.0(1.1)	1.6(1.0)	1.1(1.2)	1.2(1.3)	1.3(1.3)
Communicating health beliefs [0–4]	3.1(1.1)	2.8(1.1)	3.6(0.7)	3.0(1.1)	2.5(1.2)	2.6(1.0)	3.1(0.9)	3.6(0.9)	2.8(1.1)
Parental self-efficacy [0–4]	0.7(0.9)	0.6(0.9)	1.1(1.0)	0.6(0.9)	0.4(0.7)	0.5(0.7)	0.9(0.9)	0.4(0.7)	0.7(1.0)
Praising [0–4]	2.0(1.5)	1.7(1.3)	2.8(1.3)	1.8(1.5)	1.2(1.2)	1.6(1.2)	2.0(1.4)	2.9(1.6)	1.5(1.4)
Eating breakfast together [0–4]	3.6(2.4)	4.2(2.5)	2.3(2.2)	2.9(2.2)	5.4(2.0)	4.4(2.0)	2.9(2.0)	3.8(2.4)	4.5(2.4)

### Mediation Analyses of Associations between Family-related Variables and Children’s BMI-z-Score

#### Associations between the family-related variables and children’s BMI-z-score (c-path)

As shown in Table 3, three family-related variables were significantly associated with children’s BMI-z-score. Eating breakfast together (p<0.05) was negatively associated with BMI-z-score. Allowing to skip breakfast and negotiating about breakfast products were positively related to BMI-z-score (both p<0.05). Furthermore, no significant interactions between the different family-related variables and country of residence were found for the association with children’s BMI-z-score thus no country-specific analyses were conducted.

**Table 3:** Associations between independent (family-related factors) and dependent variable (children’s BMI-z-score), action and conceptual theory test, and mediation effects of children’s breakfast behaviour (times per week) on the association between independent and dependent variable.

Independent variables	c (SE)	c’(SE)	a (SE)	b (SE)	ab (SE)	95% CI of ab	% mediated effect
Parent behaviour(weekly breakfast) [0–7]	−0.026(0.007)	−0.017(0.007)	**0.151(0.011)** ***	−**0.061(0.009)** **	−**0.009(0.002)** *	−**0.012;** −**0.006**	
Automaticity [−2,+2]	−0.036(0.01)	−0.031(0.01)	**0.073(0.015)** *	−**0.064(0.009)** **	−**0.005(0.001)** *	−**0.007;** −**0.002**	
Availability [0–4]	0.038(0.049)	0.068(0.049)	**0.431(0.078)** *	−**0.067(0.009)** **	−**0.029(0.007)** *	−**0.042;** −**0.016**	
Encouragement [0–4]	−0.029(0.018)	−0.01(0.018)	**0.261(0.029)** **	−**0.065(0.009)** **	−**0.017(0.003)** *	−**0.023;** −**0.011**	
Paying attention [0–4]	−0.004(0.018)	0.015(0.018)	**0.232(0.029)** **	−**0.067(0.009)** **	−**0.016(0.003)** *	−**0.021;** −**0.010**	
Allowing to skip breakfast [0–4]	**0.11(0.017)** *	**0.08(0.018)** *	−**0.637(0.025)** ***	−**0.052(0.009)** *	**0.033(0.006)** *	**0.022; 0.045**	29.3
Negotiating [0–4]	**0.057(0.012)** *	**0.055(0.012)** *	−0.019(0.019)	−**0.065(0.009)** **	0.001(0.001)	−0.001; 0.004	
Communicating health beliefs [0–4]	−0.003(0.015)	−0.003(0.015)	0.013(0.024)	−**0.065(0.009)** **	−0.001(0.002)	−0.004; 0.002	
Parental self-efficacy [0–4]	0.027(0.018)	0.004(0.018)	−**0.436(0.028)** ***	−**0.064(0.009)** **	**0.028(0.004)** *	**0.019; 0.036**	
Praising [0–4]	−0.024(0.011)	−0.028(0.011)	−0.053(0.017)	−**0.066(0.009)** **	0.003(0.001)	0.001; 0.006	
Eating breakfast together [0–4]	−**0.037(0.007)** *	−**0.028(0.007)** *	**0.142(0.01)** ***	−**0.058(0.009)** *	−**0.008(0.001)** *	−**0.011;** −**0.005**	22.7

(n = 6374 child-parent dyads).

* p<0.05,

** p<0.01,

*** p<0.001.

c- coefficient: estimate of the association between family environmental item and BMI-z-score.

c’-coefficient: estimate of the association between family environmental item and BMI-z-score, adjusted for children’s breakfast consumption (mediator).

a -coefficient: estimate of the association between family environmental item and children’s breakfast consumption (mediator).

b -coefficient: estimate of the association between children’s breakfast consumption (mediator) and children’s BMI-z-score, adjusted for family environmental item.

ab product-of-coefficient estimate; mediated effect.

95% CI of ab 95% confidence interval of the mediated effect.

Three-level regression models were conducted: children nested within schools nested within countries, all regression models were adjusted for children’s gender and parent’s age, education and parent’s BMI.

#### Associations between the family-related variables and children’s breakfast consumption (a-path)

Almost all family-related variables except three (negotiating, communicating health beliefs, and praising), were strongly related with children’s breakfast consumption. Two variables (allowing skipping breakfast, and parental self-efficacy) were negatively associated with children’s breakfast consumption (p<0.001). The other six variables (parental breakfast behaviour (p<0.001), automaticity (p<0.05), availability (p<0.05), encouragement (p<0.01), paying attention (p<0.01), and eating breakfast together (p<0.001)) were positively associated with children’s breakfast consumption (see also table 3). Additionally, no interactions between the different family-related variables and country of residence were found for the association with children’s breakfast intake; consequently, no country-specific analyses were conducted.

#### Associations between children’s breakfast consumption (mediator) and BMI-z-score (b-path)

Children’s breakfast consumption was negatively related to children’s BMI-z-score (all p<0.001) (see also table 3).

#### Mediation effects (ab)

Children’s breakfast consumption mediated the associations between almost all of the family-related variables and children’s BMI-z-score except for negotiating, communicating health beliefs, and praising.

Although breakfast mediated the association between two predictors (permissiveness, and family breakfast frequency) and BMI-z-score, breakfast consumption did not fully mediate the associations, as the direct path (c’ coefficient) was also statistically significant. MacKinnon and colleagues29 stated that that the statistical significance of the c’ coefficient is a test for whether there is a complete or partial mediation. If the c’ coefficient is statistically significant and there is a significant mediation, then there is evidence for partial mediation.

#### Direct associations (c’ - path)

Some family-related variables (permissiveness, negotiating, family breakfast frequency) showed a significant direct association with the outcome (c’-path), i.e., the association between the predictor and the outcome remained significant after adjustment for the mediator (children’s breakfast consumption). This existence of a significant direct path indicates partial mediation by the proposed mediator and, thus, also designates that other factors and/or behaviours affect the association between the family-related variable and children’s BMI-z-score. A direct association between the family-related factors and children’s BMI-z-score without other significant mediating variables explaining this relation is unlikely since family-related factors are expected to affect their children’s weight status indirectly through the formation of certain eating behaviours.

## Discussion

The findings indicate that three (permissiveness, negotiating about breakfast, and family breakfast frequency) of the 11 studied family-related factors were associated with children’s zBMI. These associations, except for negotiating, were partly mediated by children’s breakfast frequency. Most of the studied family-related factors were related to breakfast consumption. However, it is likely that these family-related factors -including the physical home environment, parents’ modeling behaviour, and restrictive or supportive parental practices- are not behaviour specific but instead are indicators of a general feeding style. For example, parents who are strict with respect to breakfast consumption are likely to be strict with respect to other eating behaviours as well. The studied family-related factors may be proxies for a more general feeding style that is also related to other energy balance-related behaviours which in turn may also be related to BMI/overweight. This argument is supported by earlier research stating that that parental feeding styles can be deduced from specific food-related parenting practices. [30], [31] Hughes and colleagues [30], [31] have narrowed the definition of general parenting styles to focus solely on parenting styles related to child feeding behaviours. According to these authors, caregivers’ approach to maintain or modify children’s eating behaviours can be classified as having an authoritative, authoritarian, indulgent or uninvolved child-feeding style based on their use of demanding or responsive child-feeding behaviours and attitudes. [30]–[32] The application of the parenting style conceptualization to the feeding context implies that parents possess overarching styles that can describe how they interact with their children during all feeding situations. [32] In addition, outcomes of parenting practices may vary as a function of the general parenting style. Moreover, general parenting can moderate the association between parenting practices and children’s health outcomes, i.e. parenting styles moderate the effect of specific parenting practices because they can both positively or negatively influence the effectiveness of these parenting practices. [34] However, according to the theory of Costanzo and Woody, [33] parents do not have a single, consistent general parenting style. These authors suggested that general parenting styles differ within parents, across domains of child’s development, and across children within the same family. Costanzo and Woody [33] proposed that the extent to which parents control their children’s eating is prompted by perceptions and concerns regarding their child’s risk for obesity. Additionally, the relation between the family-related factors and BMI can also be moderated by other -more distal- factors (i.e., socio-economic status, ethnicity). [35], [36] Thus this partial mediation can be explained by the fact that overweight and obesity have a variety of causes. Moreover, it is unrealistic to expect that only a single behaviour is completely accountable for the relation between family-related variables and overweight. Breakfast consumption was not a significant mediator of the relation between negotiating and zBMI, due to the non-significant association between negotiating and children’s breakfast intake. In contrast, we found that children’s breakfast consumption was a significant mediator of the relation between availability, encouragement, paying attention and parental self-efficacy and zBMI notwithstanding the non-significant total association (c-path). According to MacKinnon, [28] a significant total association is not necessary for mediation to occur. The existence of mediation in the absence of a total association may be due to unmeasured variables that suppress the association with children’s BMI-z-score. To our knowledge, there are no previous studies that examined the mediating effect of children’s breakfast intake on the associations between family-related factors and children’s BMI-z-score.

Most family-related variables (parental breakfast behaviour, automaticity of having breakfast, availability of breakfast products, encouragement, attentiveness, permissiveness, parental self-efficacy, and eating breakfast together) were significantly associated with breakfast intake. Parental permissiveness about breakfast skipping was negatively associated with breakfast intake. This finding is congruent with previous studies that found evidence of a positive relation between more restrictive parenting practices and daily breakfast consumption. [37], [38] In our study, both physical (availability) and emotional (encouragement) support were positively related to children’s breakfast intake. However, only the relation between availability and breakfast frequency is supported by earlier research. [38], [39] Similar to previous studies, [40], [41] paying attention to what type of breakfast products are eaten by the children was positively related to breakfast intake in our study. In addition, we found that parental modelling (parental breakfast intake) and having breakfast with their parents was positively associated with children’s breakfast intake, which was also in agreement with other studies. [17], [38], [42] New findings of this study include the positive association between parents’ automaticity of eating breakfast daily and their children’s breakfast consumption and the inverse relation between children’s weekly breakfast intake and parental self-efficacy to cope with children’s nagging to skip breakfast. Children’s breakfast consumption was not associated with praising and negotiating about breakfast, and communicating health beliefs. The links between breakfast intake and both negotiating and praising were already explored in previous studies, and in line with our study as Vereecken and colleagues [37] found no significant associations.

Consistent with previous studies, [3]–[9] an inverse association between children’s regular breakfast consumption and zBMI was found. Thus, our study adds more evidence to the earlier finding that children who consume breakfast on a regular basis are likely to have a lower BMI, and are therefore at a lower risk for obesity compared with those who skip breakfast. [3]–[9] Nevertheless, as already mentioned, whether breakfast as such is of great importance, or whether skipping breakfast is an indicator of an unfavourable nutrition profile remains unclear. Next to breakfast frequency, poor breakfast quality might also influence children’s BMI. No conclusions could be drawn about the relations between breakfast quality, children’s BMI and the family-related variables based on our study since breakfast content was not included. However, earlier research confirmed the poor nutrient intake of children at breakfast. [43], [44] Thus future studies should focus on both breakfast frequency and quality when taking into account the associations with family-related factors and BMI of children.

Research results have repeatedly indicated that breakfast skipping habits are associated with a higher likelihood to be overweight or obese among school-aged children and that many schoolchildren skip breakfast occasionally or repeatedly [3]–[13]. Further exploration of potential determinants of breakfast habits and breakfast skipping is necessary to gain further insight into this issue and to possibly inform interventions. The family is expected to be of crucial importance for dietary behaviours, including breakfast habits in this age group. However, few studies have investigated potential family-related correlates of children’s breakfast consumption and the link with children’s zBMI. Moreover, no earlier studies examining the relations between a large range of family-related factors and both children’s breakfast intake and BMI-z-score are available in a large international sample. [17], [18] Thus, this study adds to the current literature concerning the association between family-related factors, children’s breakfast consumption and overweight. In addition, this study is also the first to examine the mediating effect of children’s breakfast consumption on the relations between family-related factors and children’s zBMI. Other strengths of this study are the large sample of children and parents from different European countries, the use of a standardised protocol for data collection and data processing, and the objective measurements of weight and height. However, there are some limitations. First, because this study was cross-sectional, making statements about the causality of associations was not possible. Furthermore, there were differences in response rates at student and parental levels between countries, which could have reduced the generalisability of the findings. In addition, the measurements of dietary behaviours, family-related factors and parental height and weight are based on self-report and therefore might be responded to in a socially desirable way. Moreover, to limit the burden for the participants, single items were used to measure the family-related variables which could increase measurement error. Nevertheless, the included measures showed good test-retest reliability and construct validity. [22], [23] Furthermore, earlier research showed that correlates measured with 1-item questions showed significant associations with EBRBs. [45] Another limitation of this study might be the use of parental report about the family-related factors as children’s and parents’ report could be quite discrepant. However, earlier research indicated that parents’ report of their own behaviours and the family environment may be more valid than children’s report. [39] An additional limitation of this study is the low variability in the breakfast intake score of the children.

Based on the findings, we can conclude that the family is importantly associated with both children’s breakfast behaviours and zBMI because three and seven of the 11 family-related factors were related to children’s breakfast and BMI-z-score, respectively. In particular, a focus on negotiation and rules concerning children’s breakfast consumption, parental praise for breakfast eating and family breakfast frequency may be necessary if interventions promoting breakfast eating and preventing obesity are considered because these factors were associated with both breakfast behaviour and zBMI. Nevertheless, more studies, preferably of a longitudinal and interventional nature, are needed to provide more evidence for associations between family-related factors and children’s breakfast consumption and zBMI. In addition, children’s breakfast consumption appears to be a mediator of the relations between two of the family-related factors and children’s zBMI. However, because only partial mediation was found, future studies should also focus on increasing insight into other diet-related parenting practices and general feeding styles, as well as their direct and indirect influences on children’s overweight.
